# Phytochemical screening and evaluation of *Monechma ciliatum* (black mahlab) seed extracts as antimicrobial agents

**Published:** 2013

**Authors:** Murtada Ahmed Oshi, Abdelkarim Mohmmed Abdelkarim

**Affiliations:** 1 *Departmentof Pharmaceutical Technology, Faculty of Pharmacy, Omdurman Islamic University, Omdurman, Sudan*

**Keywords:** Bacteria, Extract, Fungi, *Monechma ciliatum *Seeds

## Abstract

**Objective**: Tribes in Nubia Mountains regions of Sudan used *Monechma ciliatum *seeds for common cold and other chest allergic conditions as a traditional medicine. The aim of this paper is to validate this traditional practice scientifically.

**Materials and Methods:** *Monechma ciliatum* seeds were screened for major phytochemical groups using standard methods. Different extracts were bioassayed *in- vitro* for their bioactivity to inhibit the growth of pathogenic bacteria and fungi.

**Results:** Phytochemical screening results showed the presence of flavonoids, tannins, triterpens, and anthraquinones. *Staphylococcus* *aureus *was found to be sensitive to both water extract with zones of inhibition 22 – 26 mm at concentrations of 50 and 100mg/ml and ethanol extract 17 mm at concentration of 100 mg/ml. The growth of* Klebsiella pneumoniae *was inhibited by ethanol extract with zones of inhibition equal to 16, 26, and 33 mm at concentrations of 50, 100, and 150 mg/ml, respectively. *Pseudomonas aeruginosa *was insensitive to all extracts used*. *Similarly, all used fungi were found to be insensitive to extracts used. The minimum inhibitory concentrations of the extracts against microorganisms were ranged from 12.5 to 25 mg/ml.

**Conclusion:** The findings of the current study support the traditional uses of the plant's seed in the therapy of respiratory tract infections caused by *Staphylococcus* *aureus* and *Klebsiella* *pneumoniae*.

## Introduction

According to World Health Organization, interest in traditional systems of medicine and, in particular, herbal medicines, has increased substantially in both developed and developing countries over the past two decades (Okunlola et al., 2003 ▶).

**Figure 1:**
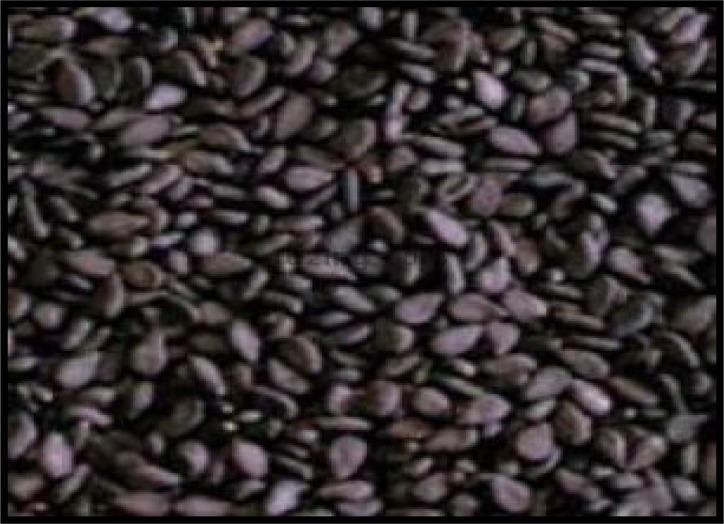
The seeds of *Monechma ciliatum*

Infections remain the main cause of morbidity and mortality in man, particularly in underdeveloped areas where it is associated with poverty and overcrowding. Common respiratory tract infections are influenza which is caused by *influenzae* virus and its main complication is secondary bacterial infection by *Staphylococcus aureus *with mortality of up to 20%, pneumonia caused by *Staphylococcus aureus, Pseudomonas aeruginosa*, *Klebsiella pneumoniae*, or anaerobic organism, and sinusitis (Kumar and Clark, 2003 ▶). Deep fungal infections have increased rapidly in recent years. Most fungal infections are caused by Candida germ, mainly *Candida albicans*. The death rate from these infections can reach 30-60%. Unfortunately, few anti-fungal medicines are available for treating fungal infections, not to mention that most of them have serious side effects (Sheng et al., 2012 ▶). 

*Monechma ciliatum* (Jacq) belongs to *Acanthaceae *family and is distributed throughout tropical Africa as wild and cultivated in savanna. It is a small herb that grows a few inches above the ground and the leaves are simple, measuring about 4–7 X 1–2 cm. In Sudan, it is locally known as 'El-Mahlab El-Aswad' in Arabic language. It grows in western and southwestern parts of Sudan especially in the Nubia Mountains and Gabel Mara area (Uguru and Evan, 2002 ▶). The seeds (Figure 1) are used as an effective laxative (Mariod et al., 2009 ▶). The seeds of the plant contain hydrocarbons and fatty acids (Ayoub, 1981 ▶). Recently, the seeds were found to contain fatty acids (e.g., palmitic, stearic, and linoleic), tocopherols, proteins, and elements (Potassium, Calcium, Magnesium, Aluminum, Lead, Nickel, Manganese, Copper, Chromium, Cobalt, and Iron) (Mariod et al., 2009 ▶).

The seeds of the plant are used as an effective remedy for common cold and other chest allergic conditions by natives in Nubia Mountains regions. The oral and inhaled traditional preparations are prepared widely by local healers to treat such conditions (personal communication). 

Successful strategies for investigation of herbal medicines involve the selection of test crude extracts based on a combination of ethnopharmacology and daily healer’s practices. Therefore, the extracts used in this research are closely resembled to traditional preparations used by natives in Nubia Mountains regions. 

## Materials and Methods

**Materials**

*The Plant Material*

The seed samples were collected from Nubia Mountains region (South Kordofan state, Sudan) between September and December (autumn season). The seeds were identified at the Department of Pharmacognosy, Faculty of Pharmacy, Alnaileen University, Sudan, and voucher specimen (No 31) was deposited at the Department of Pharmacognosy, Faculty of Pharmacy, Omdurman Islamic University, Sudan. The seeds were separated, dried under shedding, and stored in a covered bottle at room temperature until required for use. 

**Microorganisms**

*Standard bacteria and fungi*

Three types of standard bacteria namely *Staphylococcus aureus *(ATCC 25923),* Klebsiella pneumoniae *(ATCC **53657),** and* Pseudomonas aeruginosa *(ATCC 27853) were obtained from Medicinal and Aromatic Plant Research Institute (MAPRI). Three types of standard fungi namely *Aspergillus flavus *(ATCC13073), *Aspergillus niger *(ATCC 9763), and* Candida albicans *(ATCC 7596) were obtained from MAPRI.

**Preparation of the extracts**

*Maceration method using water, methanol, or ethanol as a solvent system*

The dried seeds were ground into a powder using mortar and pestle and sieved through mesh No. 35. Twenty g of the coarse powder was weighed and transferred into flasks containing 400 ml of distilled water, methanol 70% (v/v) and ethanol 70% (v/v), separately. The flasks were allowed for 24 h at room temperature, with occasional shaking. Then, the extract of each flask was passed through a cotton wool filter and the solid residue (marc) pressed (screw press). The strained and expressed liquid obtained were mixed, put for 12 h for clarification and filtrated by Whatman filter paper No 42 (125 mm). Finally, the filtrates were evaporated using rotary evaporator under reduced pressure at 40 ºC, placed in a Petri dish, and left to dry to constant weight.

*Infusion method*

The dried seeds were ground into a powder using a mortar and pestle and sieved through mesh No. 35. Twenty g of the coarse powder was weighed, transferred to two flasks, and 400 ml of cold and hot boiled water added to two flasks, separately. The flasks were shaken for a few minutes at room temperature. Then, the extract of each flask was passed through a cotton wool filter and the solid residue (marc) pressed (screw press). The strained and obtained expressed liquid was treated as above. 

*Decoction method*

The dried seeds were ground into powder using a mortar and pestle and sieved through mesh No. 35. Twenty g of the coarse powder was weighed, transferred to 4 flasks containing 400 ml of boiled water, and allowed to stand for 5, 10, 15, and 20 min, respectively. Then, the extract of each flask was passed through a cotton wool filter and the solid residue (marc) pressed (screw press). The strained and obtained expressed liquid was treated as above.

*Soxhlet extraction method by using methanol, ethanol, or chloroform as a solvent system*

The dried seeds were ground into powder using a mortar and pestle and sieved through mesh No. 35. Twenty g of the coarse powder was weighed, transferred to three Soxhlet extractor containing 400 ml of methanol 70% (v/v), ethanol 70% (v/v), and chloroform, separately, and operated for 10 h. Then, the extract of each flask was passed through a cotton wool filter and the solid residue (marc) pressed (screw press). The strained and obtained expressed liquid was treated as above.

**Preparation of the extracts**

The dry- water and ethanol extracts were separately redissolved in sterile dimethyl sulfoxide** (**DMSO) and serial dilutions of 25, 50,100, and 150 mg/ml were prepared.

**Preparation of Standard drugs**

Gentamicin and nystatin were selected as standard drugs. Serial dilutions of gentamicin (0.005, 0.01, 0.02, and 0.04 mg/ml) and nystatin (0.0125, 0.025, and 0.05 mg/ml) were prepared by using sterile DMSO as solvent.

**Preparation of standard bacterial and fungal suspension**

The bacteria were grown in Mueller Hinton agar and Mueller Hinton and broth. The fungi were grown in Sabouraud dextrose agar and Sabouraud dextrose broth. The concentration of bacterial suspensions were adjusted to 10^8^ cells/ml, and that of fungal suspensions to 10^7^ cells/ml. The turbidity of the homogenous suspensions was adjusted to approximately 0.5 Mc Farland Standard for antimicrobial activity assay (Lopez et al., 2003 ▶). 

**Testing the extract for antibacterial activity**

Antibacterial activity was studied by an agar diffusion method. The bacteria were grown overnight at 37 °C in Mueller-Hinton broth. One ml of standardized inoculum of each test bacteria was inoculated on 20 ml of molten Mueller-Hinton agar, homogenized, and poured into sterile Petri dishes. The Petri dishes were allowed to solidify inside the laminar hood. Standard cork borers of 10 mm in diameter were used to make uniform wells into which 0.1 ml plant extract dissolved in sterile DMSO was added. Plates were then incubated at 37±1 °C for 24 h. The sensitivities of the test organisms to the extract were indicated by clear zones of growth inhibition around the well containing the extracts and the diameter of the clear zone was taken as an index of the degree of sensitivity (Jonathan and Fasidi, 2003 ▶). Gentamicin and DMSO in different concentrations were used as the positive and negative controls, respectively. Inhibition zones of both were determined after incubation at 37±1 °C for 24 h.

**Testing of extract for antifungal activity**

Antifungal activity was studied by agar diffusion method. The fungi were grown overnight at 37 °C in Sabouraud Dextrose broth. One ml of standardized inoculum of each test fungi was inoculated on 20 ml of molten Sabouraud dextrose agar, homogenized, and poured into sterile Petri dishes. The Petri dishes were allowed to solidify inside the laminar hood. Standard cork borers of 10 mm in diameter were used to make uniform wells into which 0.1ml plant extract dissolved in sterile DMSO was added. Plates were then incubated at 37±1 °C for 48 h. The sensitivities of the test organisms to the extract were indicated by clear zones of growth inhibition around the well containing the extracts and the diameter of the clear zone was taken as an index of the degree of sensitivity (Hassan et al., 2011 ▶). Nystatin and DMSO in different concentrations were used as the positive and negative controls, respectively. Inhibition zones of both were determined after incubation at 37±1 °C for 48 h. 

**Minimum inhibitory concentration**

The Minimum inhibitory concentration (MIC) of the extracts was determined using the method described by (Fabry et al., 1996 ▶) with some modification. Solutions containing reconstituted extracts (50, 25, 12.5, 6.25, and 3.125 mg/ml) were incorporated into sterilized pre-poured medium of Mueller-Hinton agar (20 ml), the medium poured, and the agar in the Petri dishes was allowed to set. The Petri dishes were then inoculated with the test bacteria (*Staphylococcus aureus* and *Klebsiella* *pneumoniae*) and incubated at 37 °C for 24 h. Control Petri dishes, which contained no plant extracts, were also made with the test. The MIC of each plant was determined after 24 h (lowest concentration at which no visible growth was observed). 

**Phytochemical screening test**

Phytochemical screening was carried out on powdered specimens of the seeds using standard procedures to identify the constituents as described by (Sofowora, 1993 ▶). 

## Results

Each extraction method gave an extract with different organoleptic properties and yield percentage as presented in Table 1.

**Table 1:** The yield percentage and some organoleptic properties of extract

Extraction method	Yield %	Color	Odor	Taste	Consistency
**Maceration by water **	9.05	yellow	aromatic	bitter	solid
**Maceration by methanol 30% (v/v) **	5.15	brown	aromatic	bitter	semisolid
**Maceration by methanol 50% (v/v) **	5.45	brown	aromatic	bitter	semisolid
**Maceration by methanol 70% (v/v) **	7.40	brown	aromatic	bitter	semisolid
**Maceration by ethanol 30% (v/v) **	2.45	brown	aromatic	bitter	semisolid
**Maceration by ethanol 50% (v/v) **	5.00	brown	aromatic	bitter	semisolid
**Maceration by ethanol 70% (v/v) **	5.15	brown	aromatic	bitter	semisolid
**Infusion by cold water **	6.50	yellow	aromatic	bitter	solid
**Infusion by hot water **	9.50	brown	aromatic	bitter	solid
**Decoction 5min **	7.15	brown	aromatic	bitter	solid
**Decoction 10min **	12.13	brown	aromatic	bitter	solid
**Decoction 15min **	12.60	brown	aromatic	bitter	solid
**Decoction 20min **	13.05	brown	aromatic	bitter	solid
**Soxhlet by chloroform **	3.5	white	odorless	tasteless	liquid
**Soxhlet by methanol 70% (v/v) **	9.35	brown	aromatic	bitter	semisolid
**Soxhlet by ethanol 70% (v/v) **	6.00	brown	aromatic	bitter	semisolid

The phytochemical screening results of the seeds of *Monechma ciliatum *(Table 2) illustrated the presence of flavonoids, tannins, triterpens and unsaturated sterol.

Extensive experimental work was carried out to select the most effective extract (maceration, infusion, decoction, and Soxhlet) for antimicrobial activity test against Gram positive bacteria, Gram negative bacteria, and fungi, particularly those which cause respiratory tract infections. The results were summarized in table 3 and the effectiveness of the extract was interpreted in commonly used terms: sensitive, intermediate, and resistant and confirmed by minimum inhibitory concentrations test (Table 4).

**Table 2:** The general phytochemical test results

**Test No.**	**Detected Phytochemical**	**Result**
**1**	Alkaloids	_
**2**	Anthraquinones	++
**3 **	Coumarins	_
**4**	Cyanogenic glycosides	_
**5**	Flavonoids	++
**6**	Saponins	_
**7**	Tannins	+
**8 **	Triterpenes	+++
**9**	Unsaturated sterols	+++

(+++): very high, (++): high, (+): average, and (-): trace amout.

Water extracts (maceration) resulted in extracts with many limitations and drawbacks, as the high susceptibility to fermentation that took place after 24 h. It gave positive results against *Staphylococcus aureus* and *Klebsiella pneumoniae *as illustrated in Table 3. In ethanol extracts (Maceration), ethanol 70% (v/v) was selected for antimicrobial test due to its high extraction yield (Table 1) and for economical factors (ethanol has low cost and more availability than methanol). Ethanol 70% (v/v) gave positive results against *Klebsiella pneumoniae *and *Staphylococcus aureus* (Table 3). In infusion method, the hot infusion gave high extraction yield compared with the cold one (Table1). The hot infusion did not give results as shown in table 3. In decoction method, decoction for 20 min was superior for its high extraction yield (Table 1). It did not give positive results as shown in Table 3.

**Table 3:** Comparative bioactivity of different extracts, antibiotics, and dimethyl sulfoxide against different standard bacteria and fungi

Extraction method	Solvent system	Conc. mg/ml	Standard bacteria			Standard fungi		
			Sa Kp Pa			Ca An Af		
**Maceration**	water	25	11	-	-	-	-	-
**(24h)**		50	22	-	-	-	-	-
		100	26	-	-	-	-	-
		150	-	20	-	-	-	-
**Maceration**	ethanol	25	-	-	-	-	-	-
**(24h)**	70% (v/v)	50	11	16	-	-	-	-
		100	17	25	-	-	-	-
		150	11	33	-	-	-	-
**Infusion**	water	25	-	-	-	-	-	-
**(20min)**		50	-	-	-	-	-	-
		100	-	-	-	-	-	-
		150	-	-	-	-	-	-
**Soxhlet**	methanol	25	-	-	-	-	-	-
**(10h)**	70% (v/v)	50	-	-	-	-	-	-
		100	-	-	-	-	-	-
		150	-	-	-	-	-	-
**Gentamicin**		0.005	13	-	-	-	-	-
		0.01	14	12	-	-	-	-
		0.02	17	15	14	-	-	-
		0.04	18	22	16	-	-	-
**Nystatin**		0.0125	-	-	-	23	-	-
		0.025	-	-	-	16	14	-
		0.05	-	-	-	28	17	-
DMSO		500	-	-	-	-	-	-

Data are presented as mean diameter of growth inhibition zone (mm), average of three replicates, inhibition zone 15 mm: sensitive, 14-15 mm: intermediate, <15mm: resistant, -: no inhibition zone. Sa: *staphylococcus aureus*; Pa: *Pseudomonas aeruginosa*; Kp: *Klebsiella* *pneumoniae*; Ca: *Candida albicans*; An: *Aspergillus niger*; and Af: *Aspergillus flavus*.

**Table 4:** Minimum inhibitory concentration (MIC) of the water and ethanol extract against Staphyllococcus aureus and Klebsiella pneumoniae

Conc. mg/ml	Water extract		Ethanol extract	
	*(Staphylococcus aureus) (Klebsiella pneumoniae*)		*(Staphylococcus aureus) (Klebsiella pneumoniae) *	
**3.125**	+++	+++	+++	+++
**6.25**	+++	++	++	+++
**12.5 **	+++	++	++	*
**25**	*	*	*	-
50	**- **	** -**	**-**	-

(-) No growth, (+) light growth, (++) moderate growth, (+++) high growth, and * = MIC

*Staphylococcus aureus *was sensitive to water extract more than ethanol extract. Water extract in concentrations of 50 and 100 mg/ml gave inhibition zone diameter 22 and 26 mm, respectively, while ethanol extract gave 17 mm at 100mg/ml. Gentamicin (positive control) at concentrations of 0.01, 0.02, and 0.04 mg/ml gave inhibition zone diameter 14, 17, and 18 mm, respectively. DMSO (negative control) at concentration of 500 mg/ml did not show antimicrobial activities. Anas et al. (2008) ▶ used DMSO as solvent system for studying antibacterial activity of *Psidium guajava* Linn leaf extract. 

*Klebsiella pneumoniae *was found to be more sensitive to the ethanol extract compared with the water extract. Ethanol extract in concentrations 50, 100, and 150 mg/ml gave inhibition zone diameter 16, 25, and 33mm, respectively, while water extract was active only at 150 mg/ml with 20 mm inhibition zone. Gentamicin (positive control) at concentrations of 0.01, 0.02, 0.04 mg/ml gave inhibition zone diameter 14, 17, and 18 mm, respectively. DMSO (negative control) at concentration 500 mg/ml did not show antimicrobial activities. *Pseudomonas aeruginosa *and all used fungi were insensitive to all implemented extracts as shown in Table 3. 

The minimum inhibitory concentrations (MIC) of water extract were 25 mg/ml for both *Staphylococcus aureus *and* Klebsiella pneumoniae*, while for ethanol extract were 25 and 12.5 mg/ml for *Staphylococcus aureus *and* Klebsiella pneumonia, *respectively (Table 4)*. *The lowest MIC value of 12.5 mg/ml was obtained by ethanol extract for *Klebsiella pneumoniae. *The relatively low MIC values (12.5 and 25 mg/ml) against these two bacteria means that the seeds have the potential to treat any ailments associated with these bacterial pathogens effectively.

## Discussion

Different extraction technique gave an extract with different organoleptic properties and yield percentage. For obtaining the highest quantity and quality of phytochemical, it is necessary to know the proper methods of extraction and drying (Fathi and Sefidkon, 2012 ▶). The plant seeds contain flavonoids, tannins, and terpenoids. 

Medicinal plants have been used for centuries in folk medicines as remedies for human diseases and many active antimicrobial agents isolated form it include alkaloids, phenolic acids, quinones, tannins, coumarins, flavonoids, and terpenoids (Ahmed et al., 2006 ▶). Ethanol was better solvent. It is evident from the results that the ethanolic extract more active than aqueous extract, suggesting that the active phytochemicals are more soluble in ethanol and is the appropriate solvent for the extraction of the bioactive principles present in *Monechma ciliatum,* Tatiya et al. (2011) ▶ reported that hydroalcoholic solvents were better solvents for effective of polyphenolic compounds (tannins, flavonoids) as compared to solvents like water, methanol and ethanol.

The resistance of *Pseudomonas aeruginosa* to extracts may be due to its permeability barrier afforded by its outer membrane lipopolysaccharide and its tendency to colonize surfaces in the biofilm form which makes the cells impervious to therapeutic concentrations of antibiotics (Omolola, 2011 ▶). The insensitivity of all used fungi in the experiment to the extracts may be due to their unique cell wall. Unlike the bacteria, fungi are eukaryotic and have rigid cell walls containing chitin as well as polysaccharides and a cell membrane composed of ergosterol. Therefore, fungal infections are generally resistant to antibiotics used in the treatment of bacterial infections (Harvey and Champe, 2000 ▶). 

The crude extract of the seeds was chosen for the test instead of isolated compound due to the probability of synergistic action. The crude extracts of plants are pharmacologically more active than their isolated active principles due to the synergistic effects of various components present in the whole extract (Padmanabhan et al., 2012 ▶).

Monechma ciliatum seeds in this study exerted antibacterial activity against Staphylococcus* aureus* and *Klebsiella pneumoniae* associated with respiratory tract infections including pneumonia. The study therefore provides the scientific basis for its traditional application as ethnomedecine. The plant seeds can therefore be used for the treatment of respiratory tract infections including pneumonia. Further investigations of its activity against a wider range of bacteria and fungi and toxicological investigations of the extracts are strongly recommended.
